# Associations between negative emotions and eating behaviors in older adults: a network analysis and the mediating role of physical activity

**DOI:** 10.3389/fpubh.2025.1677170

**Published:** 2025-11-11

**Authors:** Xiuzhuan Yue, Xueying Wang, Laibing Lu, Chang Hu

**Affiliations:** 1 School of Physical Education, Henan Institute of Science and Technology, Xinxiang, China; 2 Department of Physical Education, Henan Institute of Technology, Xinxiang, China; 3 School of Physical Education and Sport Science, Fujian Normal University, Fuzhou, China; 4 Physical Education College, Jiangxi Normal University, Nanchang, China

**Keywords:** negative emotions, eating behaviors, older adults, physical activity, network analysis, mediation effect, emotional eating, aging population

## Abstract

**Objective:**

Negative emotions are a growing public health concern among older adults, influencing both psychological well-being and daily behaviors. This study aimed to examine how negative emotions relate to eating behaviors in older adults and to test the mediating role of physical activity.

**Methods:**

Data were collected from 1,062 older adults in China through validated self-report measures. A network analysis was conducted to identify central nodes and bridging indicators between negative emotions and eating behaviors, and a mediation analysis was applied to evaluate the indirect role of physical activity.

**Results:**

At the domain level, depression and stress emerged as the most central symptoms, while eating behavior showed the strongest bridging effect. At the item level, irregular eating times (Y1), perceiving life as meaningless (X20), and difficulty relaxing (X5) were highly influential. Mediation analysis indicated that physical activity partially mediated the association between negative emotions and eating behaviors.

**Conclusion:**

These findings provide new evidence that negative emotions not only directly but also indirectly shape eating behaviors in older adults by reducing physical activity. Promoting physical activity may help buffer the detrimental impact of negative emotions on eating habits and improve overall well-being. Longitudinal research is recommended to confirm the robustness and generalizability of these findings.

## 1 Introduction

Global population aging has brought increasing attention to mental health in later life. Negative emotions, including anxiety, depression, and stress, have become a major public health concern among older adults (1–5). These emotions not only compromise psychological well-being but also affect eating behaviors, which include both food choices and habitual dietary patterns (6–8). Psychological states that are common in later life, such as anxiety, depression, and loneliness, can alter dietary practices in ways that may undermine long-term health and quality of life (9, 10). The relationship between negative emotions and eating behaviors also varies across cultural and social contexts (11, 12). For instance, during the COVID-19 lockdown in Italy, heightened psychological stress among older adults was associated with unhealthier dietary habits, including more frequent snacking and greater consumption of fast foods (13). In the United States, similar patterns have been reported (14, 15), where anxiety and depression were linked to lower diet quality and inadequate nutrient intake (16). In China, by contrast, despite rapid population aging, empirical evidence on this issue remains limited. The China Report on the Nutrition and Chronic Disease Status of Residents (17), indicates that more than 190 million people aged 60 and above are living with chronic diseases, while mental health problems such as depression are increasingly common. Considering both the size of the older adults population and the significant physiological and psychological changes that occur in later life, it is critical to examine the links between negative emotions and eating behaviors in order to support health promotion and reduce long-term risks.

### 1.1 The relationship between negative emotions and eating behaviors in older adults

According to emotion regulation theory (18), individuals regulate their emotional states through various strategies. Such regulation enables adaptation to diverse environments and fulfilling psychological needs (19, 20). However, when negative emotions such as anxiety, depression, and stress become chronic, an individual’s capacity for emotion regulation may be compromised, thereby influencing behavioral decisions, including eating behaviors (21, 22).

Empirical studies provide evidence of this link. Ye et al. (23) identified loneliness, prolonged screen time, and body dissatisfaction as risk factors for emotional eating among older adults in Northeast China, while physical activity acted as a protective factor. Narchi et al. (24) reported that lower energy intake was associated with negative emotions toward food, with individuals consuming less expressing greater doubt, unease, disappointment, and indifference. Ljubičić et al. (25) found that emotional eating was linked to stress, depression, loneliness, and boredom, and motivated by weight control, the need to stay alert, and the desire to feel good. Evers et al. (26) further demonstrated that individuals with restrictive eating patterns tended to increase food intake under negative emotions, whereas positive emotions generally promoted increased consumption across all groups. Hawash et al. (27) showed that emotional eating was common among older adults, with perceived stress serving as a key determinant of their eating behavior.

Most existing research has examined the relationship between negative emotions and eating behaviors through correlational analyses (28, 29). While these methods contribute valuable insights, they largely capture linear associations and fail to account for the complex item-level interactions underlying this relationship (30, 31). Network analysis provides a promising alternative by mapping variables as nodes and their interrelationships as connections within a network (32, 33). This approach is capable of uncovering non-linear associations and intricate interaction pathways, thereby offering more nuanced insights than traditional statistical methods (34). In the context of negative emotions and eating behaviors, network analysis can identify key nodes that play dominant roles, as well as bridging indicators that connect distinct clusters of variables and reveal potential mechanisms of influence (35, 36). Previous studies have shown that this method can highlight crucial determinants and inform the development of effective intervention strategies (37, 38). Consequently, applying network analysis to the study of negative emotions and eating behaviors among older adults is of critical importance. Identifying central variables and bridging pathways can guide the design of targeted interventions, mitigate the adverse impact of negative emotions on eating behaviors, and ultimately promote healthier aging outcomes.

### 1.2 The mediating role of physical activity

As suggested by previous research, network analysis offers a broader understanding of the complex associations between negative emotions and eating behaviors by identifying important nodes and bridging indicators (39). Additionally, there may be other underlying mechanisms in this relationship, with physical activity potentially playing a mediating role (40). Physical activity, defined as various forms of bodily movements undertaken in daily life, is considered one of the best ways to achieve healthy aging and maintain physical and psychological health (41–45).

Empirical evidence indicates a significant association between negative emotions and reduced physical activity (46–49). Negative emotional states may diminish motivation to engage in physical activity, thereby leading to lower activity levels (50–52). Such reductions not only impair physical health but also exert indirect effects on eating behaviors (53). Specifically, decreased physical activity can reduce energy expenditure, disrupt appetite regulation, and influence food choices. Individuals with lower activity levels are more likely to select energy-dense, nutrient-poor foods (54–56). These unhealthy dietary patterns can, in turn, exacerbate negative emotions, creating a vicious cycle that further undermines both eating behaviors and overall health (57, 58). Prior studies suggest a potential mediating relationship, whereby negative emotions in older adults may reduce physical activity levels, subsequently leading to unhealthier eating behaviors (59, 60).

### 1.3 Current study

Although the relationship between negative emotions and eating behaviors has been widely examined, item-level interactions among older adults remain insufficiently understood (25, 61). Traditional statistical approaches are limited in their ability to capture the dynamic and complex nature of these associations. To address this gap, the present study applies network analysis to comprehensively map and interpret the connections between negative emotions and eating behaviors. This method not only illustrates the complexity of these interactions but also helps identify central and bridging indicators that play key roles in linking these constructs. The central objective of this study is therefore to explore the internal network structure connecting negative emotions and eating behaviors among older adults.

**Figure 1:**
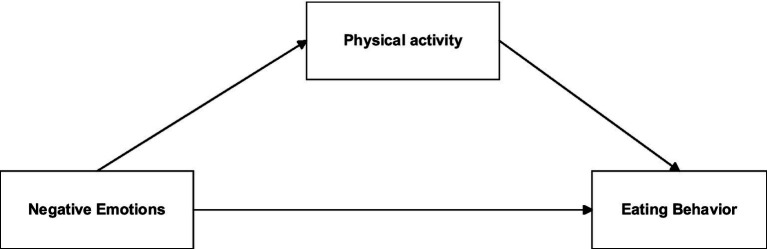
Hypothesized relationship model diagram.

In addition, considering the potential mediating role of physical activity, the study further investigates how physical activity may shape the association between negative emotions and eating behaviors. Drawing on prior evidence, we advance two hypotheses: (1) specific nodes within the network of negative emotions and eating behaviors will emerge as central and bridging symptoms; and (2) physical activity will mediate the relationship between negative emotions and eating behaviors. The hypothesized mediation model is presented in Figure 1.

## 2 Methods

### 2.1 Participants

This study was conducted between November 2024 and March 2025 using convenience and snowball sampling. Following recommended guidelines for network analysis, which require a minimum sample size of at least twice the number of estimated parameters (33), we determined that at least 400 participants were needed. The total parameter count included threshold parameters (equal to the number of nodes) and pairwise correlation parameters (calculated as nodes × (nodes – 1)/2). With 28 nodes in this study, the parameters comprised 28 thresholds and 378 pairwise correlations, yielding 406 parameters in total. Accordingly, a minimum sample size of 400 participants was required.

Participants were recruited from communities and villages in three prefecture-level cities in Henan Province, China (Xinxiang, Zhengzhou, and Jiaozuo). Inclusion criteria were: (1) age ≥ 60 years; (2) residence in the recruitment sites during the study period; (3) ability to communicate in Mandarin or the local dialect; and (4) provision of informed consent (written, witnessed oral, or proxy-based assent, as approved). Exclusion criteria were: (1) refusal to participate; (2) cognitive or intellectual impairment; and (3) prior participation in similar studies.

**Figure 2:**
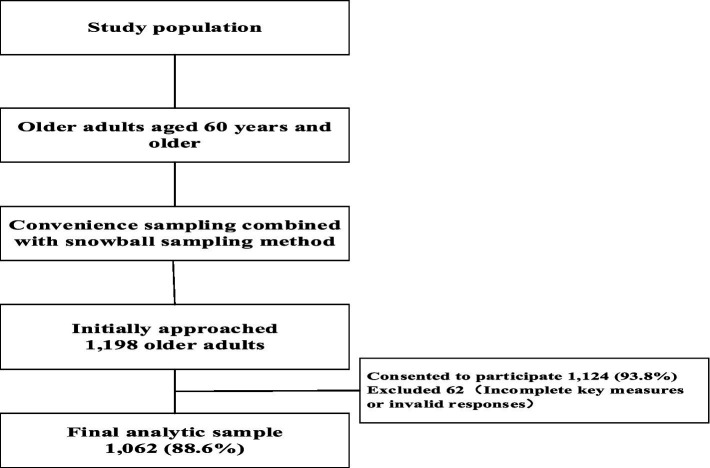
Sample flowchart.

Recruitment and response: We initially approached 1,198 age-eligible individuals across the recruitment sites. Of these, 1,124 consented to participate (overall response rate: 93.8%). After applying exclusion criteria and quality checks (e.g., incomplete key measures or patterned/random responding), 62 cases were excluded. The final analytic sample comprised 1,062 older adults (final inclusion rate among those approached: 88.6%). A CONSORT-style flowchart is shown in Figure 2.

The study adhered to the Declaration of Helsinki (62) and was approved by the Ethics Committee of Jiangxi Normal University (Approval No.: IRB-JXNU-PEC-20231108). Face-to-face interviews were conducted by uniformly trained surveyors with psychology backgrounds using ethics-approved standardized procedures. Before participation, all individuals were informed of the study purpose, procedures, risks/benefits, confidentiality, and their right to withdraw. Written informed consent was obtained from literate participants. For participants with limited literacy or who were illiterate, interviewers read the consent form verbatim in the local language, confirmed comprehension (teach-back), and obtained documented oral consent witnessed and signed by an independent witness; the ethics committee explicitly approved this oral-consent procedure. For a small subset who could not legally consent, a legally authorized representative (proxy) provided consent alongside the participant’s assent when feasible, with eligibility and procedures verified and documented on-site. All data were de-identified and securely stored with restricted access.

A total of 1,062 older adults were included in this study, with 456 males (42.9%) and 606 females (57.1%). The average age of the participants was 64.67 ± 3.11 years, ranging from 60 to 84 years. The sample was drawn from communities and villages in three prefecture-level cities (Xinxiang, Zhengzhou, and Jiaozuo) in Henan Province. In terms of education level, 725 participants (68.3%) had a primary school education or were illiterate, 293 (27.6%) had a secondary school education, and 44 (4.1%) had a university or higher education. Regarding marital status, 124 (11.7%) were unmarried, and 938 (88.3%) were married. In terms of monthly income, 286 (26.9%) had a monthly income below 1,000 RMB, 359 (33.8%) had an income between 1,000 and 2,999 RMB, 345 (32.5%) had an income between 3,000 and 5,999 RMB, and 72 (6.8%) had an income of 6,000 RMB or above.

### 2.2 Measures

#### 2.2.1 Negative emotions

Negative emotions were assessed using the Depression, Anxiety, and Stress Scale (DASS-21), which was developed by Lovibond et al. (63). The DASS-21 has been widely applied among Chinese older adults and has demonstrated strong psychometric properties (64, 65). The instrument consists of 21 items (e.g., “I felt I was close to panic”), rated on a 4-point Likert scale ranging from 0 (“did not apply to me at all”) to 3 (“applied to me very much or most of the time”). Higher total scores indicate higher levels of negative emotions. In the original validation study, the DASS-21 demonstrated excellent internal consistency (Cronbach’s *α* = 0.91). In the present study, the scale also showed strong internal consistency, with a Cronbach’s α of 0.883.

#### 2.2.2 Eating behaviors

Eating behaviors were measured using the short version of the Sakata Eating Behavior Scale (SEB-S), originally developed by Tayama et al. (66) and later translated and validated in Chinese by Ge et al. (67). The SEB-S has demonstrated good reliability and validity in previous studies. The instrument contains 7 items (e.g., “Like oily foods”), each rated on a 4-point Likert scale ranging from 1 (“strongly disagree”) to 4 (“strongly agree”). Higher scores indicate more problematic eating behaviors. In the original validation study, the SEB-S showed good internal consistency (Cronbach’s *α* = 0.83). In the present study, the scale demonstrated excellent internal consistency, with a Cronbach’s α of 0.904.

#### 2.2.3 Physical activity

Physical activity was assessed using a single self-report item: “In the past 7 days, how many days did you engage in at least 20 min of physical exercise or activity that made you sweat or breathe heavily?” For analysis, physical activity was operationalized as the number of days per week, ranging from 0 to 7, and treated as a continuous variable. Although this measure does not capture detailed variations in exercise duration or intensity, it provides a pragmatic and efficient assessment in large-scale surveys, as demonstrated by Waasdorp et al. (68). This approach has also been validated in prior health behavior research (69, 70).

### 2.3 Data analysis

Data preprocessing was first conducted. Missing data accounted for less than 1% of the dataset. To handle these values, K-means imputation was applied following established protocols (71, 72), as this approach helps preserve cluster structure and minimizes bias when missingness is low. Robustness checks were performed by comparing results with multiple imputation, yielding substantively unchanged inferences.

To explore the complex associations between negative emotions and eating behaviors, network analysis was conducted in R (version 4.3.0) using the qgraph package (version 1.9.5) with the EBICglasso algorithm (73). This analysis enabled visualization of the network structure and identification of key connections. Strength centrality was calculated via the centralityPlot function (74). To further ensure robustness, bridge strength, edge weight accuracy, and centrality stability were examined using the networktools (version 1.5.0) and bootnet (version 1.5.1) packages (75). Additional tables and figures are provided in the Supplementary materials.

To assess the mediating role of physical activity, correlation and mediation analyses were conducted. Descriptive statistics (means, standard deviations, skewness, and kurtosis) and bivariate correlations were performed using SPSS 26.0. Mediation analysis was carried out with the PROCESS macro (Model 4) (76), applying 5,000 bootstrap resamples to generate 95% confidence intervals and evaluate whether physical activity mediated the association between negative emotions and eating behaviors.

## 3 Results

### 3.1 Common method bias analysis

Because the data were collected through self-report questionnaires, Harman’s single-factor test (77) was conducted to examine potential common method bias. The analysis extracted four factors with eigenvalues greater than 1, with the first factor accounting for 26.92% of the total variance. As this proportion was below the commonly accepted threshold of 40% (78), common method bias was not considered a significant concern in the present study.

### 3.2 Network analysis of negative emotions and eating behaviors

The domain-level network of negative emotions and eating behavior dimensions comprised 4 nodes and 6 non-zero edges. Within this network, depression and stress displayed the highest node strength, whereas eating behavior showed the strongest bridge strength. Network stability was satisfactory (CS = 0.674, 95% CI [0.617, 0.776]).

At the item level, the network contained 27 nodes and 180 non-zero edges. Items Y1 (“Eat at all different times”), X20 (“I felt that life was meaningless”), and X5 (“I found it difficult to relax”) exhibited the greatest node strength, with Y1 also emerging as the most influential bridge node. Stability for this item-level network was acceptable (CS = 0.546, 95% CI [0.437, 0.626]).

**Figure 3:**
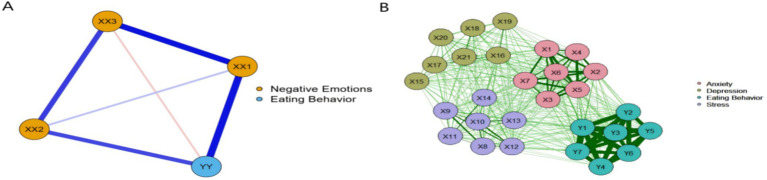
EBICglasso model based on the domain-level **(A)** and the item-level **(B)** network analysis.

These results are depicted in Figure 3. Supplementary materials provide additional details, including mean scores, standard deviations, as well as strength centrality and expected influence values for each individual item.

### 3.3 Mediation effect analysis

#### 3.3.1 Preliminary analysis

Descriptive statistics and bivariate correlations are presented in Table 1. Negative emotions were significantly negatively correlated with physical activity and significantly positively correlated with eating behaviors. Physical activity was also significantly negatively correlated with eating behaviors.

**Table 1:** Descriptive statistics and correlation analysis.

Variable	M ± SD	Skewness	Kurtosis	1	2	3
1. Negative Emotions	0.90 ± 0.49	1.527	3.806	1		
2. Physical Activity	4.13 ± 1.87	−0.338	−0.654	−0.224^**^	1	
3. Eating Behavior	2.08 ± 0.77	0.935	−0.362	0.388^**^	−0.432^**^	1

^**^p < 0.01.

#### 3.3.2 Mediation analysis

Given the significant correlation between gender and eating behaviors, gender was included as a control variable in the analysis. Using the PROCESS macro (Model 4) with standardized variables, negative emotions predicted lower physical activity (*β* = −0.223, *p* < 0.001) and higher eating behaviors (*β* = 0.302, *p* < 0.001). Physical activity predicted lower eating behaviors (*β* = −0.362, *p* < 0.001).

**Figure 4:**
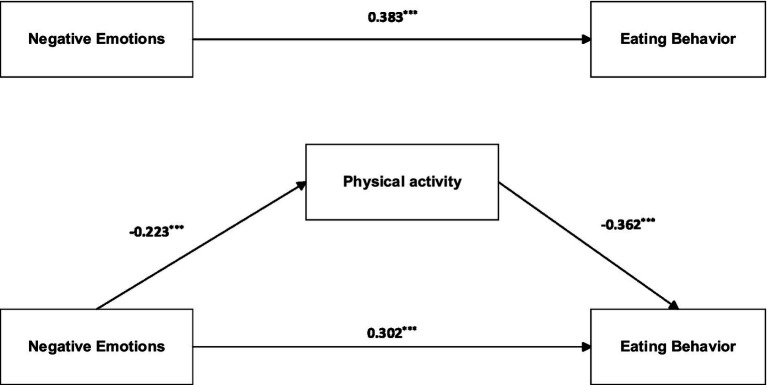
Mediation effect diagram.

Bootstrap analyses with 5,000 resamples indicated a significant total effect of negative emotions on eating behaviors (*β* = 0.383, 95% CI [0.328, 0.438]), a significant direct effect (*β* = 0.308, 95% CI [0.250, 0.354]), and a significant indirect effect through physical activity (*β* = 0.081, 95% CI [0.051, 0.105]). These findings support a partial mediation model, with 21% of the total effect mediated by physical activity. Detailed results are shown in Table 2 and illustrated in Figure 4.

**Table 2:** Mediation analysis with covariates controlled.

Variables	Physical activity		Eating behavior	
	*β*	*t*	*β*	*t*
Gender	−0.026	−0.428	0.236	4.514^***^
Negative Emotions	−0.223	−7.455^***^	0.302	11.363^***^
Physical Activity			−0.362	−13.619^***^
*R* ^2^	0.050		0.290	
F	28.054^***^		143.95^8***^	

^***^p < 0.001.

## 4 Discussion

Building on prior research, this study employed network analysis to explore the complex associations between negative emotions and eating behaviors among older adults at both the domain and item levels. In addition, we examined the mediating role of physical activity. Key nodes were identified, including depression and stress at the domain level, and specific items such as irregular eating times (Y1), feeling that life was meaningless (X20), and difficulty relaxing (X5) at the item level. Mediation analysis further indicated that physical activity partially mediated the association between negative emotions and eating behaviors. Taken together, these findings enhance our understanding of how negative emotions relate to eating behaviors in older adults and shed light on potential underlying mechanisms.

### 4.1 The relationship between negative emotions and eating behaviors in older adults

Our findings revealed a significant association between negative emotions (including anxiety, depression, and stress) and eating behaviors in older adults, consistent with prior studies (8, 23, 79). Specifically, higher levels of negative emotions were linked to more unhealthy eating patterns, such as irregular eating times (Y1), feelings of meaninglessness in life (X20), and difficulty relaxing (X5). Along with depression and stress as domain-level nodes, these items emerged as bridging indicators between negative emotions and eating behaviors. This suggests that negative emotions may disrupt the regularity of eating in older adults, thereby exerting adverse effects on overall health.

In comparison to Ye et al. (23), who identified loneliness, screen time, and body dissatisfaction as risk factors for emotional eating, our study further uncovered that depression (X20: feeling that life is meaningless) and anxiety (X5: difficulty relaxing) directly link negative emotions to eating behaviors. These two items may be associated with activating the brain’s reward circuitry, which could help explain why some older adults seek immediate pleasure through high-calorie foods (80–82). This interpretation aligns closely with the neurobiological mechanisms of emotional eating described by Evers et al. (26). Unlike earlier studies that largely relied on linear regression, our network analysis highlighted irregular eating times (Y1) as a potential indicator of the connection between negative emotions and health outcomes. Physiological research shows that circadian rhythm disruption induced by emotional disorders can directly impair metabolic health and further aggravate physiological imbalances through the temporal dysregulation of eating behaviors (83–85), lending support to our findings.

To our knowledge, this is the first study to emphasize the central role of depression and stress, together with their associated items, in the network linking negative emotions and eating behaviors using a network analysis perspective. By elucidating how specific emotional symptoms and eating patterns are interconnected, these findings refine our understanding of potential intervention targets. They underscore the importance of addressing not only the emotional states of older adults but also the regularity of their eating behaviors. By identifying critical bridging indicators, the present study offers empirical and theoretical grounding for the design of targeted interventions aimed at mitigating the negative interplay between negative emotions and eating behaviors, thereby contributing to the promotion of overall health in older adults.

### 4.2 The mediating role of physical activity

This study revealed that physical activity partially mediated the relationship between negative emotions and eating behaviors among older adults, consistent with previous research (86–88). Specifically, negative emotions were correlated with lower levels of physical activity, which in turn may be linked to more unhealthy eating behaviors, possibly forming a cyclical pattern (58, 89, 90). Lower physical activity levels were also associated with reduced energy expenditure and appetite regulation. They may contribute to a tendency among some older adults to choose high-calorie, low-nutrient foods (91, 92). The results showed that physical activity is a potential mediating factor between emotional state and eating behaviors. A decrease in physical activity levels can increase the irregularity of eating behaviors, thereby affecting overall health (93, 94). This study highlights the importance of increasing physical activity to improve older adults’ emotional state and eating behaviors. Enhancing physical activity may help regulate emotions and encourage healthier food choices, potentially interrupting the unhealthy eating patterns caused by emotional eating.

In addition, the observed negative association between physical activity and unhealthy eating behaviors indicates that increasing activity could reduce the likelihood of maladaptive eating patterns. Taken together, these findings highlight the dual role of physical activity in both regulating emotions and promoting healthier food choices. This mediating effect provides theoretical support for targeted interventions, suggesting that public health strategies should prioritize enhancing physical activity to simultaneously improve emotional well-being and eating behaviors in older adults, thereby contributing to the promotion of overall health.

### 4.3 Limitations and future directions

This research acknowledges several limitations that warrant consideration. First, the cross-sectional design prevents definitive causal inferences. Longitudinal studies that track changes in emotional states, physical activity, and eating behaviors over time are needed to clarify these factors’ directionality and dynamic interplay (85). Second, the assessment of physical activity was constrained. It relied on a single-item question, which, although practical for large-scale surveys, does not capture the activity’s frequency, duration, or intensity. This limited precision may have attenuated the observed associations. Future research would benefit from adopting validated multi-item scales or device-based measures, such as accelerometers or smartwatches, to obtain more comprehensive and accurate activity data. Third, all key variables were derived from self-reported questionnaires, susceptible to recall bias, social desirability, and random responding. Triangulation with objective indicators—such as digital food diaries, meal-timing logs, or wearable devices—would enhance both reliability and validity of data collection. Finally, the non-probability sampling strategy, combining convenience and snowball methods in three cities of Henan Province (Xinxiang, Zhengzhou, and Jiaozuo), may have introduced selection bias and limited the sample’s representativeness. Employing stratified or multistage probability sampling across broader regions and reporting detailed response rates would strengthen external validity and improve the generalizability of findings.

Notwithstanding these limitations, this study makes significant theoretical and practical contributions. It is pioneering in applying network analysis to uncover the intricate connections between negative emotions and eating behaviors, offering a novel perspective on mitigating the adverse effects of negative emotions on older adults’ eating behaviors. From a practical standpoint, identifying bridging indicators—such as Y1 (irregular eating times), X20 (feeling that life is meaningless), and X5 (difficulty relaxing) at the item level, and depression and anxiety at the domain level—highlights key areas for targeted interventions. These indicators underscore the critical role of emotional state in influencing eating behaviors and overall health. Public health initiatives and community programs should prioritize strategies for emotion management and healthy eating to counteract the negative impact of negative emotions on eating behaviors.

Furthermore, given the significant role of physical activity in modulating the relationship between negative emotions and eating behaviors, it is essential to develop interventions focused on increasing physical activity and enhancing emotional well-being among older adults. This can be effectively accomplished through community-based programs and strategies designed to foster healthy living. Additionally, educational initiatives that promote healthy eating and active lifestyles can significantly contribute to reducing the adverse effects of negative emotions on the eating behaviors of older adults.

Beyond these practice-oriented recommendations, the findings also carry broader policy implications. Developing community physical activity programs tailored to older adult populations, for example, walking groups, light exercise classes, or recreational clubs, can simultaneously strengthen physical health, bolster emotional resilience, and enhance social connectedness. Equally important are structured meal planning interventions delivered through community centers or older adult care institutions to encourage regular dietary routines and healthier food choices. At the national level, integrating such initiatives into broader frameworks such as Healthy Aging and Healthy China 2030 provides an opportunity to build a systemic, multidimensional approach that links emotional well-being, dietary regulation, and activity promotion. Such policies not only mitigate immediate risks of unhealthy eating behaviors but also foster more sustainable and resilient aging models. Moreover, these strategies hold global relevance: other rapidly aging societies can draw on these insights to inform their public health responses to the intertwined challenges of emotional health, nutrition, and aging.

## 5 Conclusion

Using both network analysis and mediation analysis, this study provides a nuanced understanding of the complex associations between negative emotions and eating behaviors in older adults. The results indicate that depression and anxiety function as pivotal indicators at the domain level. At the item level, irregular eating times (Y1), feelings of meaninglessness in life (X20), and difficulty relaxing (X5) emerged as key indicators. In addition, physical activity was found to partially mediate the relationship between negative emotions and eating behaviors. Overall, this study not only clarifies the underlying mechanisms linking negative emotions and eating behaviors in older adults but also offers valuable implications for preventing related health problems and developing targeted intervention strategies.

## Data Availability

The original contributions presented in the study are included in the article/Supplementary material, further inquiries can be directed to the corresponding author.
