# Supplementary material for: Associations between negative emotions and eating behaviors in older adults: a network analysis and the mediating role of physical activity
Source: Front Public Health. 2025 Nov 11;13:1677170. doi: 10.3389/fpubh.2025.1677170 (PMC12643853; doi:10.3389/fpubh.2025.1677170)

Supplementary Material

***Table S1.*** *mean scores, standard deviations for per item in the network.*

| item | M | SD |
| --- | --- | --- |
| X1(I found it hard to wind down) | 0.922 | 0.893 |
| X2(I tended to over-react to situations ) | 0.919 | 0.916 |
| X3(I felt that I was using a lot of nervous energy) | 0.928 | 0.888 |
| X4(I found myself getting agitated) | 0.903 | 0.893 |
| X5(I found it difficult to relax) | 0.914 | 0.914 |
| X6(I was intolerant of anything that kept me from getting on with what I was doing) | 0.923 | 0.894 |
| X7(I felt that I was rather touchy) | 0.887 | 0.855 |
| X8(I was aware of dryness of my mouth) | 0.921 | 0.902 |
| X9(I experienced breathing difficulty (e.g. excessively rapid breathing, breathlessness in the absence of physical exertion) | 0.864 | 0.884 |
| X10(I experienced trembling (e.g. in the hands) | 0.874 | 0.907 |
| X11(I was worried about situations in which I might panic and make a fool of myself) | 0.883 | 0.892 |
| X12(I felt I was close to panic) | 0.856 | 0.905 |
| X13(I was aware of the action of my heart in the absence of physical exertion (e.g. sense of heart rate increase, heart missing a beat) | 0.840 | 0.871 |
| X14(I felt scared without any good reason) | 0.896 | 0.902 |
| X15(I couldn’t seem to experience any positive feeling at all) | 0.862 | 0.871 |
| X16(I found it difficult to work up the initiative to do things) | 0.926 | 0.896 |
| X17(I felt that I had nothing to look forward to) | 0.924 | 0.875 |
| X18(I felt down-hearted and blue) | 0.941 | 0.894 |
| X19(I was unable to become enthusiastic about anything) | 0.895 | 0.887 |
| X20（I felt that life was meaningless.) | 0.920 | 0.901 |
| X21( I felt I wasn’t worth much as a person) | 0.936 | 0.907 |
| Y1(Eat at all different times) | 1.977 | 1.014 |
| Y2(Do not feel satisfied unless I eat until full) | 2.118 | 0.954 |
| Y3(Eat fast) | 2.098 | 0.973 |
| Y4(Tend to gain weight more easily than others) | 2.097 | 0.948 |
| Y5(Like oily foods) | 2.092 | 0.959 |
| Y6(Eat if others around me are eating) | 2.050 | 0.972 |
| Y7(When buying food, I am not content unless I buy more than necessary) | 2.134 | 0.970 |

X1-7Stress,X8-14Anxiety,X15-21Depression,Y1-7Eating behaviors

***Table S2.****Differences in eating behaviors across demographic variables*

| Variables | Demographic category | n | % | Mean (SD) | t/F value |
| --- | --- | --- | --- | --- | --- |
| Gender | Male | 456.00 | 42.90% | 1.96(0.66) | -4.51^***^ |
|  | Female | 606.00 | 57.10% | 2.17(0.84) |  |
| Educational level | Primary school or illiterate | 725.00 | 68.30% | 2.09(0.78) | 0.220 |
|  | Secondary school | 293.00 | 27.60% | 2.07(0.76) |  |
|  | College or above | 44.00 | 4.10% | 2.01(0.70) |  |
| Marital status | Single | 124.00 | 11.70% | 1.98(0.82) | -1.56 |
|  | Married | 938.00 | 88.30% | 2.09(0.77) |  |
| Monthly income | Less than 1,000 RMB | 286.00 | 26.90% | 2.10(0.80) | 0.12 |
|  | 1,000 to 2,999 RMB | 359.00 | 33.80% | 2.07(0.76) |  |
|  | 3,000 to 5,999 RMB | 345.00 | 32.50% | 2.08(0.79) |  |
|  | 6,000 RMB or more | 72.00 | 6.80% | 2.04(0.63) |  |

Note.^***^*P*<0.001

***Figure S1.*** *Accuracy of edge weights in the domain-level network.*


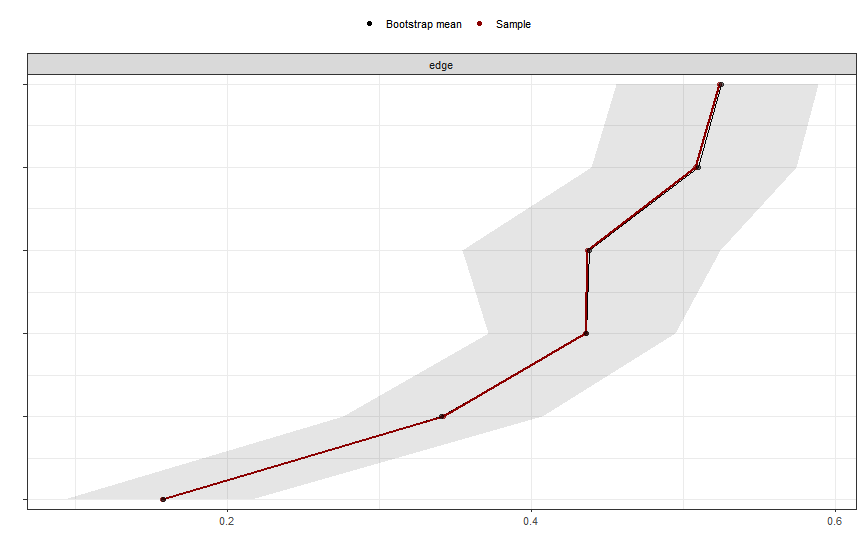


***Figure S2.*** *Accuracy of edge weights in the item-level network.*


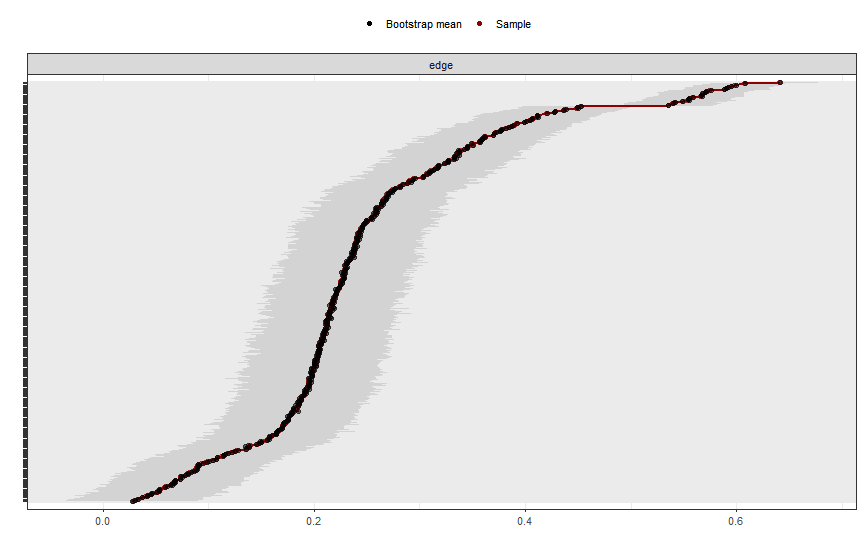


***Figure S3.*** *Stability of strength and closeness centrality in the domain-level network.*


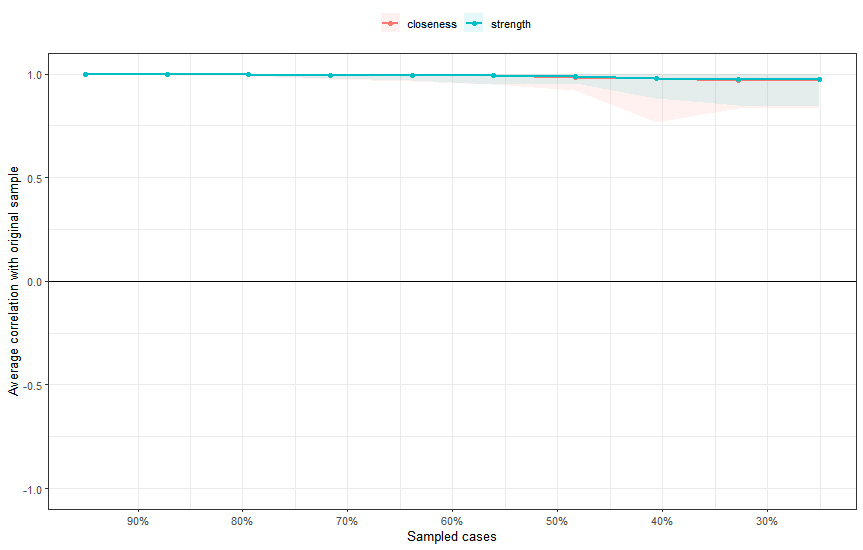


***Figure S4.*** *Stability of strength and closeness centrality in the item-level network.*


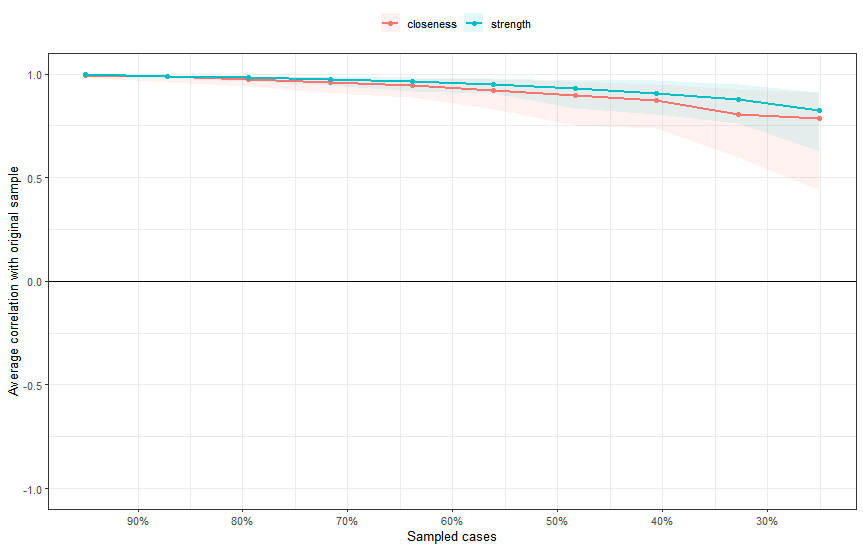


*Figure S5. Centrality plots for the domain-level network*


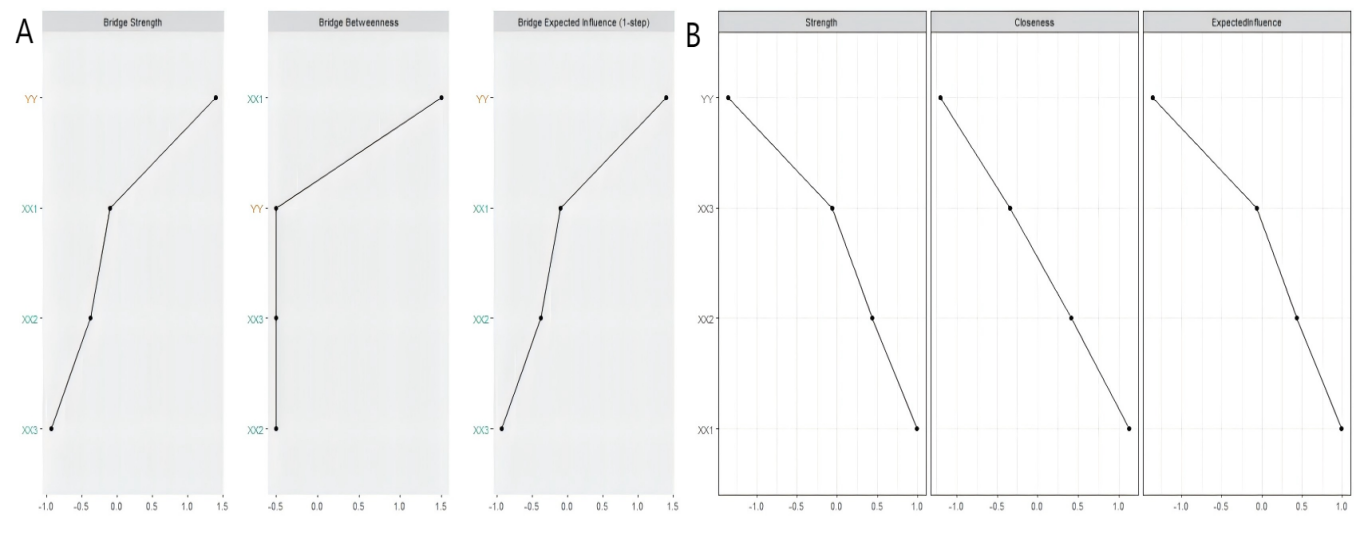


***Figure S6.*** *Centrality plots for the item-level network*


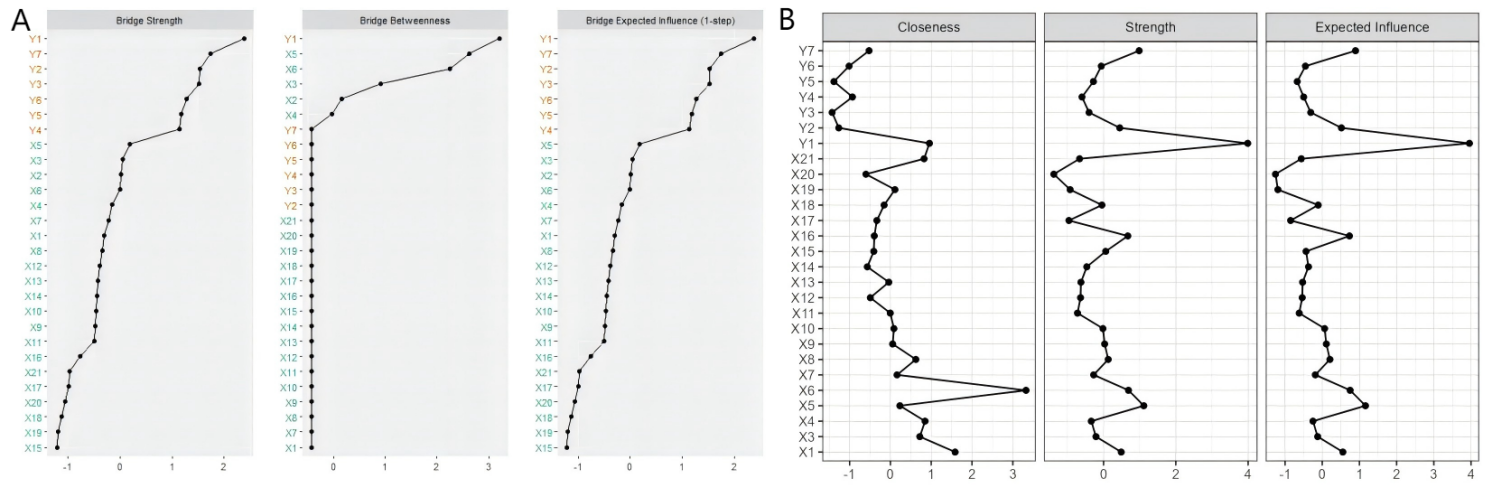


***Figure S7.*** *Normal distributions with P-P plots.*


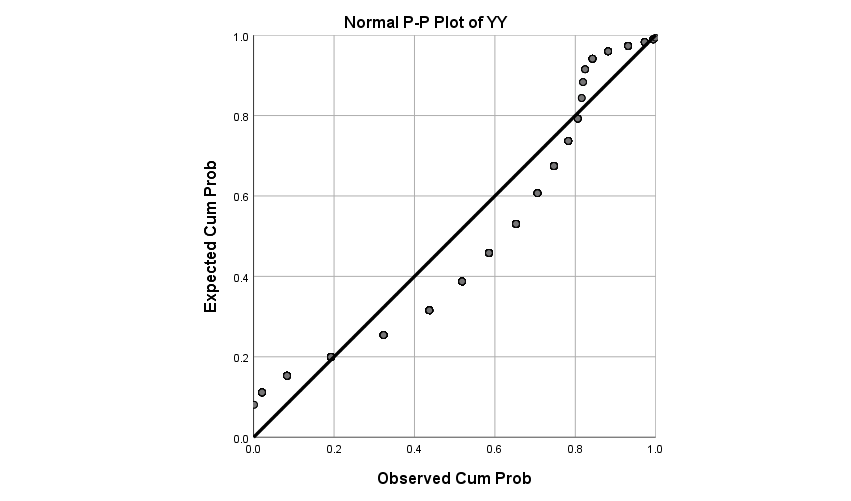

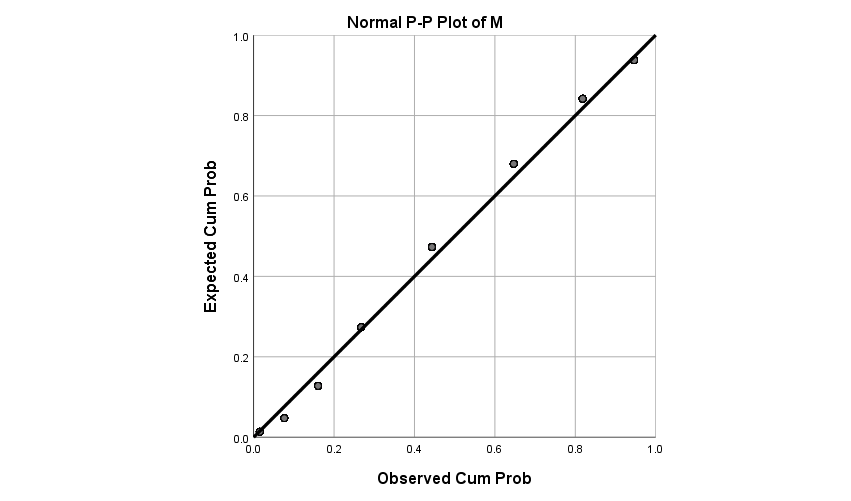

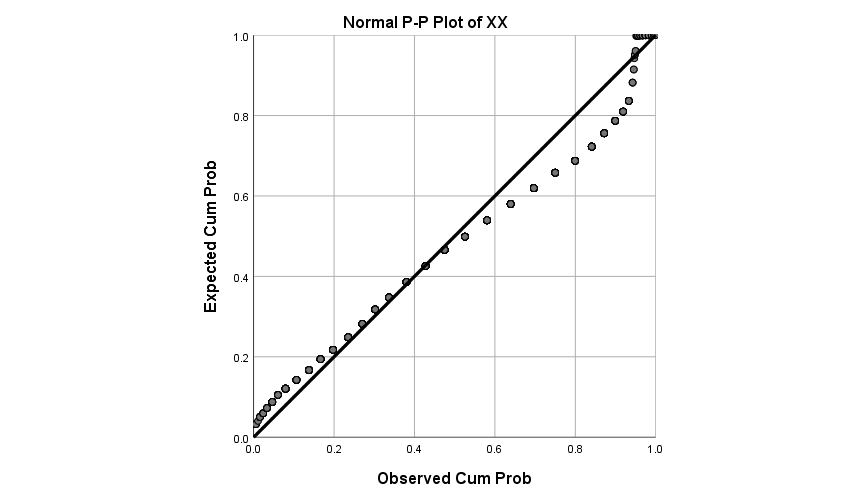

Supplement: Supplementary file 1 [file Supplementary_file_1.docx]
